# On the Causes of Dyspepsia and Other Diseases to Which Students Are Liable

**Published:** 1832

**Authors:** James C. Finley


### Art. IV.—*On the Causes of Dyspepsia and other Diseases to which Students are liable.*—By James C. Finley, M. D.

1. The most efficient cause of dyspepsia is *Want of exercise.* Man is intended to be an active, as well as a rational being, and disease is the penalty annexed by nature, to the neglect of the active duties of life.

A very cursory investigation of the effects of exercise will shew that inactivity is not only a very powerful direct cause of disease, but that by co-operating with the other causes of disease to which students are exposed, it has a more general and pernicious influence than any other, perhaps than all the rest.

Exercise by facilitating the motion of the blood in the veins, throws an increased quantity upon the heart; this organ thus stimulated to greater exertion circulates the blood more rapidly through the lungs, and consequently exposes it more frequently to the action of the air, and secures the beneficial changes which result from that contact; this increased action at the same time, impels the blood with more energy into the capillary vessels, where the various functions of secretion, exhalation, nutrition, and the generation of animal heat are carried on. Thus the circulation is promoted, and the proper balance between the different parts of the vascular system is preserved, the complexion is made to exhibit the roseate hue of health, animal temperature is regulated, sensibility is imparted to the nerves, energy to the muscles, and activity to that whole system of waste and supply, by which the healthful condition of the body is preserved.

Directly the reverse are the consequences of inactivity. The blood accumulates in the veins producing visceral obstructions and depression of spirits; it is imperfectly oxygenized and consequently the muscles are languid and debilitated, and the functions of the different organs imperfectly performed; the animal heat is diminished, and the system rendered sensible to atmospheric changes; and lastly there is a liability to those forms of disase which arise from obstructed perspiration. In addition to these circumstances, it is well known that the body, when debarred from its regular exercise, becomes irritable, and to use a familiar expression, "nervous," with a desire for unnatural stimuli, and that this condition extends its influence to the mind, rendering it peevish, fretful, impulsive, and unfit for close application to study.

It will thus be seen that, from the constitution of man, exercise is necessary to supply the defects of his organization, and, if we may use the expression, is an essential part of the animal economy; the negative influence of sedentary habits, therefore, in predisposing the body to disease, must be very considerable. This influence, however, is generally so gradual, that it is seldom fully recognized, and is not unfrequently entirely lost sight of, in the operation of causes of disease which derive all their deleterious influence from the derangement and debility consequent npon indolence; and we shall have an opportunity of remarking in the enumeration of the other causes of disease, to which students are exposed, that with a constitution strengthened by laborious exercise and habitual exposure, they would have little to apprehend from disease.

2. *Intemperance in eating.* In order to the preservation of health, there should be some ratio between the amount of food and the waste of the body. The appetite, if its dictates were attended to, would generally regulate this; but the gratification of the taste so generally leads to a disregard of the sense of satiety, and an artificial appetite is so universally provoked by the use of stimulants, that students of the most sedentary habits, very frequently devour as great a quantity of food, as the man whose body is exhausted by the most violent and long continued labor. The stomach is thus exposed to a double source of injury, viz. it is filled with food disproportionate to the wants of the system, and it is stimulated to the performance of the task of digesting this, by condiments which impart temporary power, at the expense of future debility. Dyspepsia, sooner or later, is the inevitable consequence, and its symptoms are not the less distressing, because they are accompanied with the deceptive appearance of general health, or even of an excess of nutrition.

3. *The use of unnatural stimulants.* We have already remarked that one of the tendencies of sedentary habits, was to produce a restlessness—an irritability of the body, and a consequent resort to artificial stimulants for its relief. Of these, the most common, and, with the exception of ardent spritis, the most pernicions, is tobacco in the various forms of smoking, chewing, and snuffiing. The evils of these noxious and vulgar habits are incalculable, and the more injurious because they produce their effects so insidiously, that the cause is seldom suspected until the habit has become inveterate. Men of active habits may often indulge in these practices with impunity, but to students they are always injurious, impairing the power of digestion, and increasing the irritability both of the body and of the mind.

In conformity with custom, I have called this weed a stimulant. It is however the reverse of a stimulant in every respect, directly impairing the powers of life, and not doing its work indirectly, as ardent spirits do.

4. *Studying in unnatural positions*—with the body much bent—with the feet elevated above the head—writing with the chest or stomach resting upon the edge of the table, &c.; by which the healthful play of the vital organs is obstructed, and a foundation laid for general or local diseases. Much of the disposition to these uncomfortable positions, arises from the want of that vigorous tone of the muscular system which can only be obtained by active exercise.

5. *Studying in cold or damp rooms or in wet clothes.* The inactivity of study must necessarily favour the operation of this cause of disease. The evil will be further increased by the diminished power of generating animal heat, and of resisting the impressions of morbid agents, which want of exercise produces. This latter circumstance has also a more direct influ ence in aggravating the effects of cold. We have already remarked that one of the tendencies of want of exercise was, to permit the blood to recede from the surface of the body, and to accumulate in the large venous trunks. Now this is precisely the influence of cold, and consequently, the two causes combining must produce a proportionately greater effect.

6. *The habit of studying in heated apartments.* The languor of the circulation consequent upon sedentary habits, renders the body peculiarly sensitive to cold, and hence the disposition to keep up the animal heat by an undue degree of external warmth. The immediate consequences of this are, that the skin is either too much relaxed, or that fever is produced, and the surface becomes dry and harsh, and the body restless and uncomfortable; languor and feebleness are produced, and a liability to the evils produced by atmospheric vicissitudes.

7. *Late and irregular hours of retiring to rest, and consequently of rising.* The healthful tendency of early rising, and the injurious tendency of protracting study to a late hour are proverbial. One hour devoted to sleep before midnight is said to be worth two after it. In explanation of this fact, besides the reasons generally assigned, we beg leave to offer the following physiological facts. Close application to study does not indispose the body for labor, although bodily fatigue is a serious obstacle to intellectual exertion, and renders a double effort of the attention necessary to its accomplishment. Again, while labor disposes the body to repose, mental exertion induces wakefulness, and there are few intense students whose active minds have not often caused them to pass sleepless nights, at a time when their exhausted powers imperiously demanded repose.

Nature therefore obviously indicates the morning as the period of study. The labors of the day are thus converted into healthful amusement, and in their turn serve to procure a sound and refreshing sleep at night. By inverting the order, the student sits down to study with his mind incapacitated for the occupation; mental exertion is doubly exhausting: the labors of the day are protracted, the tendency to repose is interrupted; his slumbers are disturbed, and consequently the succeeding day is one of languor and exhaustion. In confirmation of this view, I have never known an individual engaged in business of an active nature, who had accustomed himself to late hours of study, that could be induced to make a great mental effort, except on the spur of great and immediate excitement.

It may further be remarked, that the hours of late students are almost of necessity irregular. Now sleep, like almost every thing else appertaining to the animal economy, is very much under the influence of habit; and consequently, where the hours of repose are irregular, much inconvenience will frequently be the consequence.

The excuse so frequently urged by some, that night is the most favorable period for study, proves nothing but the strength of a pernicious habit.

8. *The abuse or neglect of suitable remedies.* Many students suffer from the injudicious use of medicine so powerful as to impair the healthful tone of the stomach, at a time when fasting or on abstemious diet is rather called for. The temporary relief produced by these means encourages a repitition of excesses and a recurrence to the remedy, until by their combined influence dyspepsia is produced. On the other hand the neglect to use remedies for removing certain conditions of the system to which sedentary men are predisposed very frequently lays the foundation for dyspepsia or more serious diseases.

